# Current Role of Hysterectomy in Pelvic Floor Surgery: Time for Reappraisal? A Review of Current Literature and Expert Discussion

**DOI:** 10.1155/2021/9934486

**Published:** 2021-07-06

**Authors:** Guenter K. Noé, Annelize Barnard, Sven Schiermeier, Michael Anapolski

**Affiliations:** ^1^University of Witten-Herdecke, Germany; ^2^Department of Obstetrics and Gynecology, Rheinland Klinikum Dormagen, Germany; ^3^Department of Obstetrics and Gynecology, University of Stellenbosch, South Africa; ^4^Department of Obstetrics and Gynecology, University Witten-Herdecke, Germany

## Abstract

Since hysterectomy could be performed with low risk, it has been part of the standard of surgical prolapse therapy for decades. This has not been scrutinized for a long time. In this review, we describe the development of this issue in recent years. The current literature suggests that hysterectomy requires its own indication. The article describes the various options for a uterine-preserving surgical technique and the available data.

## 1. Introduction

Most of the literature on which the article is based was researched via PubMed (2020). In addition, we have used historical book literature for the introduction. Surgical techniques for the correction of vaginal prolapse have been in use since the 19^th^ century [1]. These were aimed at the narrowing of the vaginal canal, and hysterectomy did not play an integral part in addressing prolapse. At the beginning of the 20th century, the Manchester-Fothergill operation was introduced [1]. This procedure is based on the amputation of the cervix and relocation of the suspending ligaments to the lower corpus uteri. This technique is still in use today, but unfortunately, there is limited data on complications, long-term effects, or success rates.

Other techniques, such as those described by Schauta, Wertheim, and Watkins, utilized the uterus as a support by steeply anteverting it and then sewing it to the anterior vaginal wall [1]. Like the Manchester operation, uterine interposition has long had supporters in Scandinavia. As these techniques predate rigorous modern scientific assessment, empiric data is lacking. The first sacropexy was described as early as 1920 in Germany. Either the uterus or the vault was sutured to the promontory via laparotomy.

The indications for a hysterectomy as part of a prolapse operation have repeatedly changed, as described in a German study over the period from 1960 to 1985. Only 24.3% of the prolapsed interventions were combined with a hysterectomy between 1960 and 1963, while between 1978 and 1985 97.7% of the interventions were combined with a hysterectomy [2]. The indications for the inclusion of a hysterectomy were mainly cancer prevention and birth control. The hysterectomy offered no improvement in the long-term success of the prolapse procedure. On the contrary, DeLancey stressed the importance of the paracervical structures as early as 1992 for the prevention of cystocele and rectocele [3]. Disadvantages of uterine conservation have not yet been reported. A new study from the Netherlands has investigated the sacrospinous fixation with uterine preservation versus the combination with a hysterectomy. Superiority for uterine preservation was determined [4] (follow-up after 5 years: 87% versus 76%). These results are not surprising since, for example, problems with the mesh fixation in combination with hysterectomy are known in sacropexy [5]. The study would be more meaningful if the technique had also been compared with patients who had had a previous hysterectomy.

A meta-analysis from 2018 describes generally shorter operation times, lower blood loss, and lower mesh exposure rates when the uterus is preserved. The analysis is based on 54 abstracts that compared vaginal and abdominal procedures with and without hysterectomy. Although the essential results (less operating time and blood loss) were to be expected, the analysis supports the advantage of attaching mesh or suture material to the cervix [6].

There are certainly clear indications for a hysterectomy that are medically justified. A German study group has defined the following indications and recommended them as German S3 guidelines: symptomatic fibroids or painful adenomyosis, recent or previous cervical pathology, abnormal or postmenopausal bleeding, tamoxifen therapy, familiar BRCA 1 and 2 risk, status post hereditary nonpolyposis colorectal cancer with 40-50% lifetime risk of endometrial cancer, and no regular gynecological follow-up assured [7]. These indications are however not mandatory in all cases, since hysterectomies are still possible after a prolapse operation.

A 2010 study with a cohort of 501 patients reported that the risk of missing an endometrial malignancy is approximately 0.8% [8]. Unfortunately, the cohort is too small to allow for generalization. In our own data, we found 2 endometrial cancers in 600 procedures with hysterectomy (0.03%) and none in the hysteropexy group to date. Larger studies are needed to determine the true incidence; however, 0.5% seems likely.

One important consideration should be the patient's desire. A study from 2013 investigated reasons for hysterectomy as reported by patients; 213 women were interviewed at multiple centers. Only 20% of the women desired a hysterectomy while 36% were clearly opposed to it. In the second group, a fifth would have accepted a poorer outcome, while 44% were unable to commit themselves [9]. In addition to the possibly better outcome, we currently see the desire to retain fertility and the desire to preserve the physical integrity of the body as reasons for maintaining the uterus.

Vaginal as well as laparoscopic techniques are available in many centers today. While sacropexy is considered an established practice, the study data for vaginal techniques (especially vaginal meshes) are limited. In 2013, the data of 507 women who underwent laparoscopic hysteropexy over a period of 10 years were retrospectively examined [10].

Outstanding features of the study were a low complication rate of 1.8% and no mesh exposure. The hysteropexy could not be completed in 17 patients (3.4%). A total of 93.8% of the patients stated that their prolapse was “very much” or “much” better. Only 2.8% required repeated apical surgery.

Based on the literature, one can state that the preservation of the apical structures has a positive effect on operative data and long-term results. We have already listed clear indications. The question is that are there any other indications for a hysterectomy? With an abdominal approach, a large uterus can cause technical difficulties. This relates to access to the operative field and difficulties in bringing in additional meshes. There are no data in this regard, only expert recommendations. A recommendation based on weight or size would be difficult to define, since all local conditions in the pelvis have to be taken into account. With a vaginal approach, there are fewer limitations due to the size of the uterus.

## 2. Available Techniques

### 2.1. Vaginal Techniques

For several decades, the sacrospinous ligament was used for apical fixation. Sacrospinous fixation was introduced in the 1950s [11]. It was used all over the world and was a great advancement in vaginal apical fixation. It could be combined very well with a colporrhaphy, but anatomically, it had the disadvantage that it was a unilateral suspension so that the vaginal axis shifted. A 2013 Cochrane analysis looked at randomized trials that compared vaginal (especially sacrospinous fixation) and sacrocolpopexy (SC). The review showed the superiority of SC, but also highlighted the significantly longer operating times and the longer learning curve for SC [12].

After emerging criticism of mesh surgery and severe restrictions or even bans on these technologies, the sacrospinous fixation was revived. It is still performed according to the traditional method or with the help of suturing devices to fix the sutures [13]. Numerous companies offer small meshes instead of sutures to improve the result. The meshes are fixed with sutures or anchors. Similar to the traditional procedure, the anchors or sutures are placed in the ligament close to the pudendal nerve. The execution of the techniques under direct vision is very difficult and is therefore usually done blindly under the guidance of the index finger. Therefore, good surgical skills and extensive training are necessary. Incorrect placement can result in very uncomfortable long-term consequences for the patient. So far, only relatively limited data from single-center studies are available [14, 15]. These studies report excellent results for the combination of bilateral mesh-assisted sacrospinous fixation with traditional colporrhaphy.

A review published in 2021 reports, among other things, 300 mesh-supported hysteropexies carried out in a German single center. The author states that the technique can be completed in just 22 minutes and provides excellent results. Despite the high number, unfortunately, no study has been published in this regard yet [16]. Most publications relate to short-term data with no results for long-term mesh-related complications that can arise from fibrosis or mechanical stress or irritation. The same applies to traditional methods such as the Manchester-Fothergill technique or high-uterosacral fixation. The literature search yielded a handful of small studies and case reports. Neither randomized nor prospective studies are available in published form.

The culdoplasty procedure, often referred to as the McCall technique, is used to prevent prolapse after a hysterectomy. As part of the general mesh discussion, these techniques are also recommended as native tissue apical repair techniques at conferences. This can of course also be thought of as a uterus-preserving technique. There is also no usable data in this regard. Schiavi et al. compared two suturing techniques for culdoplasty and found the preventive value of both techniques. Suspension sutures were performed in all patients in both study groups. There was no control group in the study, and it did not provide an analysis of the general risks of a pelvic floor defect. Despite these fundamental study flaws, the authors come to the conclusion that the method is effective [17]. Therefore, there is a lack of real evidence for the efficacy of these procedures as prophylaxis and they cannot be recommended as a replacement for apical fixation. A study that proves the efficacy of what is known as prolapse hysterectomy should not go unmentioned. Similar to the Manchester technique, the technique is based on the high-level integration of the uterine ligaments [18]. Even if the data are convincing, the complete lack of real long-term data, randomized studies, or multicenter applications also applies here.

### 2.2. Laparoscopic Procedures

**Figure 1:**
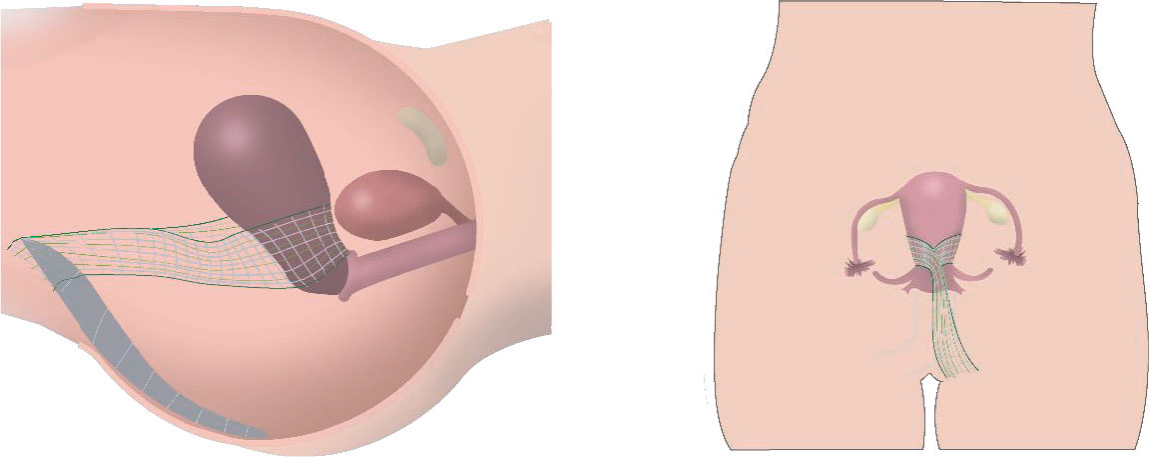
Hysterosacropexy in Oxford technique.

In laparoscopy, sacropexy dominates due to its widespread use. Very often it is combined with subtotal hysterectomies or with a total hysterectomy. For the latter, there is a higher exposure rate to consider [5]. Unfortunately, there are no case numbers on the frequencies of the procedures used. Currently, it can still be assumed that one of the forms of hysterectomy is used in the majority of cases [19]. Different procedures are described in the literature for hysteropexy. On the one hand, a mesh is only attached between the sacrum and the posterior wall of the cervix, while others carry out bilateral fixations and sew a mesh onto or through the posterior wall of the cervix [20]. An often-cited surgical approach is a method known, among others, as the Oxford technique (Figure 1). A caudally 2-armed mesh is passed through a window in the broad ligament and tied anteriorly to the cervix [21]. The cranial portion is then attached to the promontory, and the mesh is then peritonealized.

In 2016, Jefferis et al. published a 10-year follow-up with highly satisfying data. The majority of the patients had been treated in the 6 years before the evaluation (after completing the learning curve). Only 2.8% of the women had to undergo another operation and stated a high level of satisfaction. The intraoperative complication rates were also very low. The surgical method therefore seems to be very safe and successful. One weakness of the study is that the patients were not physically reexamined and the data related to returnees and records. In addition, there is a lack of randomized prospective studies or even multicenter analyses when used outside of a specialized center.

Another surgical technique that is often used is the lateral suspension. So far, there is little published study data for the procedure and no description of the hysteropexy.

**Figure 2:**
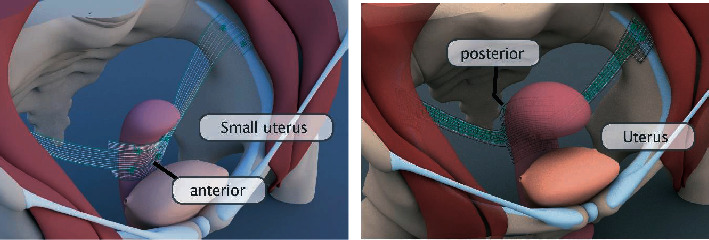
Anterior fixation and posterior fixation in hysteropectopexy with PRP 3 × 15 or PRP 3 × 18, respectively.

In 2010, the laparoscopic pectopexy was first published with a small pilot study [22]. After initial prospective randomized study, the safety of the technology in widespread use was proven by a multicenter study with 11 clinics and 13 surgeons [23]. The follow-up to this study was also able to demonstrate the high effectiveness of the technology [24]. Hysteropexy can also be performed with this technique. Small uteri can easily be fixed anteriorly to the standard mesh. For larger uteri, an extended mesh was developed (Figure 2) (DynaMesh PRP 3 × 18) which enables the uterus to be picked up dorsally. The dorsal fixation is done to prevent retroflexion. The PRP 3 × 15 can be attached directly to the uterus with a PVDF (polyvinylidene fluoride) thread without the need for peritonealization. This is possible because both the mesh and the thread are made of PVDF and thus do not provoke any adhesions. The lateral arms are passed through a small window in the broad ligament and then typically fixed laterally (Table 1 gives an overview on the current studies dealing with hysteropexy).

## 3. Conclusion

Today, it is undisputed that the hysterectomy itself does not make a significant contribution to the correction of pelvic floor defects. In fact, there are rather clear indications that the procedure is disadvantageous. Longer operating times and higher mesh exposure rates in total hysterectomy are documented. Few true indications are clear, and some are relative as described above. The influence of uterus size on abdominal procedures is unclear. The size ratio between the pelvic space and the uterus should allow a smooth operation. Ultimately, this must be decided by the surgeon and, if possible, planned ahead. Both vaginal and abdominal procedures for hysteropexy are available. Abdominal, predominantly laparoscopic surgical techniques have been scientifically proven. Some of the newer vaginal procedures are very promising, but require well-structured, scientific research, especially with regard to the mesh problem of recent years.

Surgical technique today should be resilient with regard to the skills of the surgeon. Since urogynecological interventions are carried out worldwide and not exclusively by specialists, techniques should be investigated in their broad application. This requires multicenter studies. Too many techniques are said to be simple, and even less well-trained surgeons may be tempted to perform them. This has also been one of the problems with vaginal mesh surgery. The removal of the uterus should always be subject to strict indications, and the reflex hysterectomy should be relegated to the past. As previously noted, there is a long tradition of hysterectomy as part of prolapse surgery, so research must further specify the indications.

## Figures and Tables

**Table 1:** Hysteropexy studies 2010-2021.

Author	Technique	DOI	rT	Multicenter	Single center	Prospective	Follow-up time	Outcome	*n*
Veit-Rubin	Lateral suspension	10.1007/s00192-015-2859-6	No	No	Yes	No	1 year	82.7% success	250
Yassa	Lateral suspension	10.1055/a-0941-3485	No	No	Yes	No	2 years	94.12	17
Detollenaere	Sacrospinous hysterectomy versus preservation	10.1136/bmj.h3717	Yes	Yes	No	Yes	1 year	Similar outcome in both groups	208
Lo	Sacrospinous hysterectomy versus preservation	10.1111/jog.12678	No	No	Yes	No	7 years	Similar outcome	146/26
Nager	Sacrospinous hysterectomy versus preservation	10.1016/j.ajog.2021.03.012	Yes	No	Yes	Yes	5 years	Hysteropexy with mesh is superior to hysterectomy with uterosacral suture suspension	
Gutman	Vaginal versus laparoscopic hysteropexy		No	No	Yes	No	1 year	Similar anatomical outcome vaginal mesh exposure 3 times higher	150
Price	Sacropexy	10.1111/j.1471-0528.2009.02396.x	No	No	Yes	No	3 months	High satisfaction	51
Jefferice	Sacropexy	10.1007/s00192-016-3257-4	No	No	Yes	No	3 months	92%	498
Li	Sacropexy	10.4103/tcmj.tcmj_131_19	No	No	Yes	No	1 year	Similar outcome	26
Pan	Sacropexy TLH versus hysteropexy	10.1007/s00192-015-2775-9	No	No	Yes	No	1 year	Similar outcome	99
Van IJsselmuiden	Sacropexy versus sacrospinous	10.1111/1471-0528.16242	Yes	No	Yes	Yes	1 year	Anatomical similar LSH defecation disorders and OAB more frequent, less dyspareunia	126
Letouzy	Ant mesh repair	10.1007/s00192-015-2748-z	No	No	Yes	No	1 year	92% 8% exposure	114

No data for uterus weight or other distinguishing features for indication available.

## Data Availability

All data are related to the cited references in the manuscript.
